# Pooled analysis of PCV13 efficacy from controlled human infection trials in Malawi and the UK

**DOI:** 10.1038/s41541-026-01381-4

**Published:** 2026-02-26

**Authors:** Evaristar Kudowa, Godwin Tembo, Anthony E. Chirwa, Tarsizio Chikaonda, Alfred Muyaya, Lumbani Makhaza, Edna Nsomba, Bridgette Galafa, Faith Thole, John Ndaferankhande, Lorensio Chimgoneko, Neema Toto, Dingase Dula, Ben Morton, Shaun H. Pennington, Angela Hyder-Wright, Andrea M. Collins, Elena Mitsi, Daniela M. Ferreira, Stephen B. Gordon, Marc Y. R. Henrion, EHPC consortia, Mark Alderson, Jeremy Brown, Marien L. De Jonge, David Goldblatt, Jason Hinds, Simon Jochems, Richard Malley, Christopher Bailey, Bernard Beall, Stephen Bentley, Debby Bogaert, Joe De Campo, Stuart Clarke, David Cleary, Adam Finn, Brad Gessner, Stephen Gordon, Caz Hales, Melanie Hamon, Robert Heyderman, Kondwani Jambo, Hiroshi Kiyono, Samuel Leong, Marc Lipsitch, Alex Mann, Michael Mina, Eliane Miyaji, Helder Nakaya, Giorgio Napolitani, Daniel Neill, Mihai Netea, Marco Oggioni, Peter Openshaw, Fernanda Peterson, Sue Plummer, Brenda Kwambana, Adam Roberts, Imran Saleem, Andreas Schlitzer, Alex Shalek, Timothy Tobery, John Tregoning, Jeffrey Weiser

**Affiliations:** 1 Malawi Liverpool Wellcome Research Programme, Blantyre, Malawi; 2 Department of Statistics, University of Pretoria, Pretoria, South Africa; 3 Liverpool School of Tropical Medicine, Liverpool, UK; 4 Liverpool University Foundation Trust, Liverpool, UK; 5 Oxford Vaccine Group, Department of Paediatrics, University of Oxford, Oxford, UK; 6 Centre for Inflammation Research, University of Edinburgh, Edinburgh, UK; 7 Center for Vaccine Innovation and Access, PATH, Seattle, Washington USA; 8 ImmunoBiology Ltd., Babraham, Cambridge, United Kingdom; 9 Centre for Inflammation Research, Edinburgh Medical School, University of Edinburgh, Edinburgh, United Kingdom; 10 Division of Medicine, University College London, London, United Kingdom; 11 Clinical Research Department, London School of Hygiene & Tropical Medicine, London, UK; 12 Laboratory of Medical Immunology, Radboudumc Center for Infectious Diseases, Radboud University Medical Center, Nijmegen, The Netherlands; 13 Department of Clinical Sciences, Liverpool School of Tropical Medicine, Liverpool, UK; 14 Great Ormond Street Institute of Child Health Biomedical Research Centre, University College London, London, UK; 15 Centre for Genomics and Child Health, Blizard Institute, Queen Mary University of London, London, UK; 16 Research Department of Infection, Division of Infection and Immunity, University College London, Rayne Institute, London, United Kingdom; 17 Institute for Infection and Immunity, University of London, St. George’s, United Kingdom; 18 Department of Parasitology, Leiden University Medical Center, Leiden, Netherlands; 19 Research Center for Advanced Science and Technology, The University of Tokyo, Tokyo, Japan; 20 Boston Children’s Hospital and Harvard Medical School, Boston, MA USA; 21 Department of Medicine, Kamuzu University of Health Sciences, Blantyre, Malawi; 22 Department of Public Health, School of Public and Global Health, Kamuzu University of Health Sciences, Blantyre, Malawi; 23 The Ethox Centre, Nuffield Department of Population Health, University of Oxford, Oxford, UK; 24 Department of Medicine, Queen Elizabeth Central Hospital, Blantyre, Malawi; 25 Department of Mathematics and Statistics, University of Strathclyde, Glasgow, UK; 26 New York University, Grossman School of Medicine, New York, USA; 27 Eagle Global Scientific, LLC, Contractor to Respiratory Diseases Branch, Centers for Disease Control and Prevention, Atlanta, Georgia USA; 28 Parasites and Microbes, Wellcome Sanger Institute, Cambridge, UK; 29 The University of Edinburgh/MRC Centre for Inflammation Research, The Queen’s Medical Research Institute, 47 Little France Crescent, Edinburgh, UK; 30 Antigen Discovery, Incorporated, Irvine, California USA; 31 Faculty of Medicine and Institute for Life Sciences, University of Southampton, Southampton, SO17 1BJ UK; 32 Bristol Children’s Vaccine Centre, Schools of Population Sciences and Cellular and Molecular Medicine, University of Bristol, Bristol, United Kingdom; 33 Pfizer Vaccines, Collegeville, Pennsylvania USA; 34 Malawi Liverpool Wellcome Programme, Blantyre, Malawi; 35 School of Nursing Midwifery and Health Practice, Faculty of Health, Victoria University of Wellington, Wellington, New Zealand; 36 Institut Pasteur, G5 Chromatine et Infection, Paris, France; 37 Department of Human Mucosal Vaccinology, Chiba University Hospital, Chiba, Japan; 38 ENT Department, Aintree University Hospital, Liverpool, UK; 39 Center for Communicable Disease Dynamics, Department of Epidemiology and Department of Immunology and Infectious Diseases, Harvard T. H. Chan School of Public Health, Boston, Massachusetts USA; 40 hVIVO Services Ltd, London, UK; 41 Department of Genetics, Harvard Medical School, Boston, MA 02115 USA; 42 e Laboratory of Bacteriology 2, Instituto Butantan, Sao Paulo, Brazil; 43 Department of Clinical and Toxicological Analyses, School of Pharmaceutical Sciences, University of São Paulo, São Paulo, Brazil; 44 Medical Research Council (MRC) Human Immunology Unit, MRC Weatherall Institute of Molecular Medicine (WIMM), John Radcliffe Hospital, University of Oxford, Oxford, UK; 45 Department of Clinical Immunology, Microbiology and Immunology, University of Liverpool, Liverpool, United Kingdom; 46 Department of Internal Medicine and Radboud Center for Infectious Diseases, Radboud University Medical Center, Nijmegen, The Netherlands; 47 Department of Genetics and Genome Biology, University of Leicester, Leicester, UK; 48 Department of Infectious Diseases, Imperial College London, London, United Kingdom; 49 Institute of Oral Biology, Faculty of Dentistry, University of Oslo, Oslo, Norway; 50 Cultech Ltd., Baglan, Port Talbot, SA12 7BZ UK; 51 Department of Parasitology, Liverpool School of Tropical Medicine, Pembroke Place, Liverpool, L3 5QA UK; 52 School of Pharmacy and Biomolecular Sciences, Liverpool John Moores University, Liverpool, L3 3AF UK; 53 Myeloid Cell Biology, Life and Medical Sciences Institute, University of Bonn, Bonn, Germany; 54 Department of Immunology, Massachusetts General Hospital, Boston, MA 02114 USA; 55 Unilever, Trumbull, CT USA; 56 Imperial College London, Department of Infectious Disease, London, UK; 57 Department of Microbiology, New York University Grossman School of Medicine, New York, New York USA

**Keywords:** Conjugate vaccines, Experimental models of disease

## Abstract

We conducted the first pooled analysis of two randomised controlled vaccine trials on experimental pneumococcal serotype 6B carriage, registered in Malawi (PACTR202008503507113) and the UK (ISRCTN45340436). This post-hoc exploratory study examined the sex-based differences in carriage, vaccine efficacy and vaccine-induced responses. PCV-13 reduced colonisation by 76% (*p* < 0.001) with non-significant interaction by sex (RR = 1.549, *p* = 0.413). Females showed a higher carriage rate than males (28% vs. 19%, *p* = 0.066). Baseline anti-6B Capsular Polysaccharide Immunoglobulin G (IgG) titres were higher in females, significantly in Malawi (2.62 µg/ml vs males 2.05 µg/ml, *p* = 0.015). Post-vaccination titres did not differ by sex. The pooled fold change in IgG pre-post vaccination, was higher in vaccinated females (5.47 vs 3.30, *p* = 0.053). This analysis demonstrates the utility and challenges of integrating CHIM data between diverse settings to evaluate vaccine efficacy, describe inter-setting differences, investigate biological and immunological factors influencing protection against pneumococcal carriage and ultimately inform future vaccine development strategies.

## Introduction

Pneumococcal disease remains a leading cause of morbidity and mortality worldwide, particularly in low- and middle-income countries (LMICs), where over 90% of pneumococcal-related deaths occur^1^. According to the World Health Organization (WHO), sub-Saharan Africa bears one of the highest burdens of pneumococcal disease, with children under 5 years and adults ≥50 years at greatest risk^2^. In low-resource settings where vaccine coverage, malnutrition or comorbidities may interact with immune responses, it is important to understand whether certain sub-groups such as those defined by sex, experience differences in vaccine protection against pneumococcal disease, as this could allow more efficient targeting of improved vaccination strategies.

Increasing evidence suggests that biological sex may influence susceptibility to pneumococcal disease and immune response to pneumococcal vaccination^3–6^. Males across age groups have shown to experience higher burden of invasive pneumococcal disease (IPD) and pneumonia, even after the introduction of pneumococcal conjugate vaccines (PCVs)^7^. While sex differences in immune responses to other vaccines are well described^7–9^, with females often having stronger humoral and cellular responses, population based evidence from adult cohorts suggest possible sex-related variations in pneumococcal vaccine effectiveness^10^. However, the mechanisms underlying these differences in the context of pneumococcal carriage and vaccine protection remain underexplored.

Controlled human infection models (CHIMs), in which healthy adult volunteers are deliberately exposed to a pathogen under a carefully controlled and safe condition, have become increasingly useful tools for studying disease pathogenesis and evaluating novel vaccines^11^. These ethically approved studies involve the intentional introduction of a well-characterised pathogen strain to consenting participants to study disease mechanisms, identify correlates of protection and evaluate vaccine efficacy^11^. Advantages of CHIMs include the use of smaller sample sizes, reduced time and cost in vaccine development, the ability to generate rapid efficacy data during emerging epidemics or pandemics^12^. They are also important for studying rare infections for which animal models are inadequate or fail to replicate human immune responses^12^. By controlling the timing, dose, and pathogen strain, CHIMs allow for assessment of vaccine-induced protection against colonisation. CHIM findings also inform vaccination strategies including, dose schedules and approaches to protect the high risk population^12^.

The Malawi Accelerated Research in Vaccines by Experimental and Laboratory Systems (MARVELS) programme^13^ in Malawi and the Experimental Human Pneumococcal Carriage (EHPC) programme^14^ in Liverpool, UK, have utilised CHIMs to investigate factors associated with carriage of *Streptococcus pneumoniae*. Both programmes have evaluated the efficacy of the 13-valent pneumococcal conjugate vaccine (PCV-13) against pneumococcal carriage^14,15^ of a 6B serotype strain. Routine PCV-13 immunisation schedules differ between Malawi and the UK, with Malawi implementing a 3 + 0 schedule^16^ and the UK transitioning from a 2 + 1 to a 1 + 1 schedule^17^. Differences in vaccine schedules may influence baseline immunity and carriage dynamics, but none of the subjects in our studies had prior pneumococcal vaccination (this is an exclusion criterion). It is possible but unlikely that differing historical infant PCV schedules may have shaped baseline adult immunity through indirect exposure or natural boosting^18–20^, among unvaccinated participants, given the lack of herd immunity seen in these populations.

PCV-13 has demonstrated remarkable efficacy against IPD in Malawi with vaccine effectiveness estimated at 70–85% against vaccine-type (VT) IPD in children under 5 years of age and 62% among older people between 18 and 45 years^15,21,22^. However, despite widespread childhood vaccination, pneumococcal carriage remains significant in this setting^16^. In the UK, PCV-13 has demonstrated success in reducing invasive disease rates in young children, indirectly protecting unvaccinated populations by curbing community transmission of VT pneumococci^14^. However, its limited serotype-independent protection continues to pose challenges and there is persistent VT carriage in certain populations and the emergence of non-vaccine serotypes which affects the overall impact on carriage^14^.

Despite this progress, there are several gaps in our understanding of the impact of sex on pneumococcal carriage rates, disease development, host immune responses to vaccination and vaccine efficacy against mucosal disease, as in pneumococcal pneumonia. Identifying and understanding the sex-specific differences in pneumococcal disease dynamics has the potential to contribute to the development of targeted interventions and improvement of vaccination strategies. A study that examines the sex-specific disparities in pneumococcal carriage and vaccine efficacy with CHIMs would be informative.

We therefore utilised data from two distinct settings: Malawi (MARVELS programme) and the UK (EHPC programme), to conduct a pooled analysis focusing on pneumococcal carriage, and explore the relationship between PCV-13 vaccine efficacy, sex, and humoral capsular polysaccharide immune responses. This combined approach capitalises on the similarities in study designs, outcome definitions and challenge agents used in both programmes. This is the first pooled analysis from CHIM trial data on PCV-13 from two distinct settings, exploring sex-based differences in carriage outcomes and immune response. Malawi, a low-income country with a high burden of pneumococcal disease, high HIV prevalence, high force of infection, high urban population density and limited access to antibiotics, presents a unique context to study the impact of PCV-13 and sex in a resource-limited setting. The UK, a developed country setting with robust healthcare infrastructure, provides a contrasting perspective. By pooling data across these two distinct settings, we leverage the larger combined sample size for a broader evaluation of PCV-13 efficacy, immune responses and an interrogation of the influence of sex on pneumococcal carriage dynamics in two contrasting settings. This analysis also allows us to better investigate setting-specific differences between both study programmes. We hypothesised that PCV-13 would reduce experimental colonisation and that sex-specific differences in immune response would impact vaccine efficacy.

## Results

### Malawi PCV-13 CHIM study

Out of 204 participants in the Malawi study, 98 were randomised to receive PCV-13 vaccine, and 106 to receive saline placebo (Table 1; Supplementary Fig. 1). The median age of participants was 25.3 years (interquartile range, IQR: 22.9–28.5). There were 147/204 (72%) males, 75/147 (51%) in the PCV-13 arm. Pneumococcal carriage was observed in 40/204 (20%) participants, and stratified by sex, in 27/147 (18%, 95% confidence interval (CI): 13–26) males and in 13/57 (23%, 95% CI: 13–36) females (relative risk (RR) = 0.81, 95% CI: 0.45–1.45, *p* = 0.556). Among the vaccinated group, there were 10/98 (10%, 95% CI: 6–18) carriers of Spn6B; 6/75 (8%, 95% CI: 4–17) in males and 4/23 (17%, 95% CI: 7–36) in females (RR = 0.50, 95% CI: 0.15–1.63, *p* = 0.238). Clearly, this study had small numbers, but the sex difference in vaccinated subjects raised a concern about higher carriage rates and hence reduced vaccine efficacy in females. Among the unvaccinated group, there were 30/106 (28%, 95% CI: 21–37) experimental carriers with 21/72 (29%, 95% CI: 20–41) in males and 9/34 (27%, 95% CI: 14–43) in females (RR = 1.10, 95% CI: 0.57–2.14, *p* = 0.821).

**Table 1:** Demographic characteristics and carriage status by sex for MARVELS and EHPC

	MARVELS *N* = 204		EHPC *N* = 96		Overall *N* = 300	
	Female	Male	Female	Male	Female	Male
**Participants**						
*n* (%)	57 (28)	147 (72)	59 (61)	37 (39)	116 (39)	184 (61)
**Age (years)**						
Median (IQR)	25 (23, 29)	25 (23, 28)	21 (20, 24)	22 (20, 23)	23 (21, 27)	24 (22, 27)
**Vaccination status:** ***n*** **(%)**						
PCV-13	23 (40)	75 (51)	29 (49)	19 (51)	52 (45)	94 (51)
Control (Saline for MARVELS Hepatitis A (Avaxim) for EHPC)	34 (60)	72 (49)	30 (51)	18 (49)	64 (55)	90 (49)
**6B Carriage** ***n*** **(%, 95% CI)**						
Yes	13 (22.8%, 12.7–35.8)	27 (18.4%, 12.5–25.6)	20 (33.9%, 22.1–47.4)	8 (21.6%, 9.8–38.2)	33 (28.4%, 20.5–37.6)	35 (19.1%, 13.6–25.4)
No	44 (77.2%, 64.2–87.3)	120 (81.6%, 74.4–87.5)	39 (66.1%, 52.6–77.9)	29 (78.4%, 61.8–90.2)	83 (71.6%, 62.4–79.5)	149 (80.9%, 74.6–86.4)

The distribution of participants, age, and 6B pneumococcal carriage status by sex across the MARVELS and EHPC cohorts. Data are reported as counts (n) with percentages, and median age with interquartile ranges (IQR). We have included the exact 95% binomial confidence intervals (95% CI) for all proportions in the table. Carriage status is shown for serotype 6B, categorised as ‘Yes’ (carriage) or ‘No’ (non-carriage). Note that other serotypes were monitored but have been excluded from this analysis.

### UK PCV13 CHIM study

In the UK study, 48/96 participants received the PCV-13 vaccine, while 48/96 received the Hepatitis A vaccine (Table 1; Supplementary Fig. 1). The median age was 21.0 years (IQR: 20.0–24.0). There were 37 (39%) males, with 19/37 (51%) in the PCV-13 arm. Pneumococcal carriage was observed in 28/96 (29%) participants: 8/37 (22%, 95% CI: 10–38) males and 20/59 (34%, 95% CI: 22–47) females (RR = 0.64, 95% CI: 0.31–1.30, *p* = 0.251). Among the vaccinated group, 5/48 (10%, 95% CI: 5–23) were experimental carriers: 1/19 (5%, 95% CI: 1–24) males and 4/29 (14%, 95% CI: 6–31) females (RR = 0.38, 95% CI: 0.05–3.16, *p* = 0.635). Again, there was an observed higher rate of experimental carriage in vaccinated females compared to males. In the unvaccinated group, there were 23/48 (48%, 95% CI: 34–62) carriers: 7/18 (39%, 95% CI: 19–63) males and 16/30 (53%, 95% CI: 35–70) females (RR = 1.12, 95% CI: 0.55–2.25, *p* = 0.383).

### Vaccine efficacy

The log-binomial model for Malawi, adjusted for dose and sex, showed a significant reduction in carriage risk with PCV-13 (RR = 0.283, 95% CI: 0.122–0.652; *p* = 0.003). The relative risk for females compared to males was 0.818 (95% CI: 0.425–1.576; *p* = 0.549). A statistically significant reduction in carriage risk was observed in the 20,000-dose group compared to the 160,000-dose group (RR = 0.230, 95% CI: 0.058–0.915; *p* = 0.037). The interaction between vaccination status and female sex was not significant (RR = 2.409, 95% CI: 0.535–9.144; *p* = 0.196; Table 2).

**Table 2:** Log-binomial model summary for MARVELS population

	Estimate	95% CI	SE	*Z* statistic	*p*-value
(Intercept)	0.351	0.227–0.540	0.221	−4.746	<0.001
Vaccination: PCV-13	0.283	0.122–0.652	0.427	−2.958	**0.003**
Sex: Female	0.818	0.425–1.576	0.334	−0.6	0.549
Dose: CFU 20,000	0.230	0.058–0.915	0.705	−2.086	**0.037**
Dose: CFU 80,000	0.988	0.576–1.693	0.275	−0.044	0.965
PCV-13 vaccine: Female	2.409	0.535–9.144	0.681	1.292	0.196

Log-binomial regression model results for the MARVELS population, estimating the relative risk (RR) of experimental pneumococcal carriage (serotype 6B). The outcome is binary carriage status at any follow-up time point. Independent variables include vaccination status (PCV-13 vs. reference = saline), sex (female vs. reference = male), inoculation dose (20,000 CFU and 80,000 CFU, reference = 160,000 CFU), and the interaction between sex and vaccination status. Estimates represent RR values, 95% confidence intervals (CI), with accompanying standard errors (SE), Z statistics, and *p*-values. A RR < 1 indicates reduced risk of carriage. The model includes only participants from the MARVELS study (*N* = 204). Carriage status is for experimental pneumococcal serotype 6B Note that other serotypes were monitored but have been excluded from this analysis.

The bold values in the *p*-value colum are for the signifi cant p-values. All *p*<0.05 for all variables in the model.

The log-binomial model for the UK study, adjusted for sex showed a significant reduction in carriage risk with PCV-13 (RR = 0.135, 95% CI: 0.018–0.994; *p* = 0.049). Neither female sex (RR = 1.371, 95% CI: 0.703–2.677; *p* = 0.355) nor the interaction between PCV-13 and female sex (RR = 1.911, 95% CI: 0.208–17.539; *p* = 0.567) was statistically significant (Table 3).

**Table 3:** Log-binomial model summary for EHPC population

	Estimate	95% CI	SE	*Z* statistic	*p*-value
(Intercept)	0.389	0.218–0.694	0.295	−3.196	0.001
Vaccination: PCV-13	0.135	0.018–0.994	1.017	−1.966	**0.049**
Sex: Female	1.371	0.703–2.677	0.341	0.926	0.355
PCV-13 vaccine: Female	1.911	0.208–17.539	1.131	0.573	0.567

Log-binomial regression model results for the EHPC population, estimating the relative risk (RR) of experimental pneumococcal carriage (serotype 6B). The binary outcome reflects carriage status at any follow-up time point. The model includes vaccination status (PCV-13 vs. reference = control [hepatitis A vaccine, Avaxim]), sex (female vs. reference = male), and the interaction between sex and vaccination status. Reported values include the estimated relative risk (Estimate), 95% confidence intervals (CI), standard error (SE), Z statistic, and *p*-value. A RR < 1 indicates a lower risk of carriage. The total sample size for this model is 96 participants. Carriage status is for experimental pneumococcal serotype 6B. Note that other serotypes were monitored but have been excluded from this analysis.

The bold values in the *p*-value colum are for the signifi cant *p*-values. All *p*<0.05 for all variables in the model.

The log-binomial model for the pooled analysis combining data from Malawi and UK showed a significant reduction in carriage risk with PCV-13 (RR = 0.239, 95% CI: 0.100–0.519; *p* < 0.001) Table 4. Female sex was not associated with carriage risk (RR = 1.153, 95% CI: 0.740–1.798; *p* = 0.529). There was no statistically significant association with study programme (RR for EHPC = 1.462, 95% CI: 0.969–2.204; *p* = 0.070), but the borderline significance (*p* = 0.070) suggests heterogeneity in experimental carriage rates between the UK and Malawi settings. No significant interaction was observed between PCV-13 and female-sex (RR = 1.549, 95% CI: 0.543–4.418; *p* = 0.413; Table 4). Results from a sensitivity analysis adjusting for inoculation dose in the pooled model showed a similar significant reduction in carriage risk associated with PCV-13 (RR = 0.245, 95% CI: 0.113–0.528; *P* < 0.001) Supplementary Table 1. The overall carriage rate among the 80,000-dose group were 22.2% (14.9–31.8) for MARVELS and 29.2% (21.0–38.9) for EHPC Supplementary Table 2.

**Table 4:** Combined log-binomial model summary for Malawi and EHPC population

	Estimate	95% CI	SE	*Z* statistic	*p*-value
(Intercept)	0.284	0.204–0.395	0.169	−7.472	<0.001
Vaccination: PCV-13	0.239	0.100–0.519	0.396	−3.615	**<0.001**
Sex: Female	1.153	0.740–1.798	0.226	0.630	0.529
Setting: EHPC	1.462	0.969–2.204	0.210	1.812	0.070
PCV-13 vaccine:Female	1.549	0.543–4.418	0.535	−0.819	0.413

Log-binomial regression model results from the pooled analysis combining data from the MARVELS and EHPC studies. The model estimates the relative risk (RR) of experimental pneumococcal carriage (serotype 6B), with covariates including vaccination status (PCV-13 vs. control), sex (female vs. male), study site (EHPC vs. MARVELS), and the interaction between sex and vaccination. Reported estimates include the relative risk (Estimate), 95% confidence intervals (CI), standard error (SE), Z statistic, and *p*-value. A RR < 1 indicates a lower risk of carriage. The study site variable compares EHPC (UK) to MARVELS (Malawi), with MARVELS as the reference. The total sample size for the model is 300. Note that other serotypes were monitored but have been excluded from this analysis.

The bold values in the *p*-value colum are for the signifi cant *p*-values. All *p*<0.05 for all variables in the model.

### Anti capsular polysaccharide -IgG expression and sex differences

The Malawi study had 197/204 participants with both pre- and post-vaccination serum samples, and the UK study had 92/96 participants pre- and post-vaccination serum samples for Capsular Polysaccharide (CPS) -specific IgG quantification. There were 7/204 (3%) and 4/96 (4%) missing paired samples for Malawi and UK studies respectively.

**Fig. 1:**
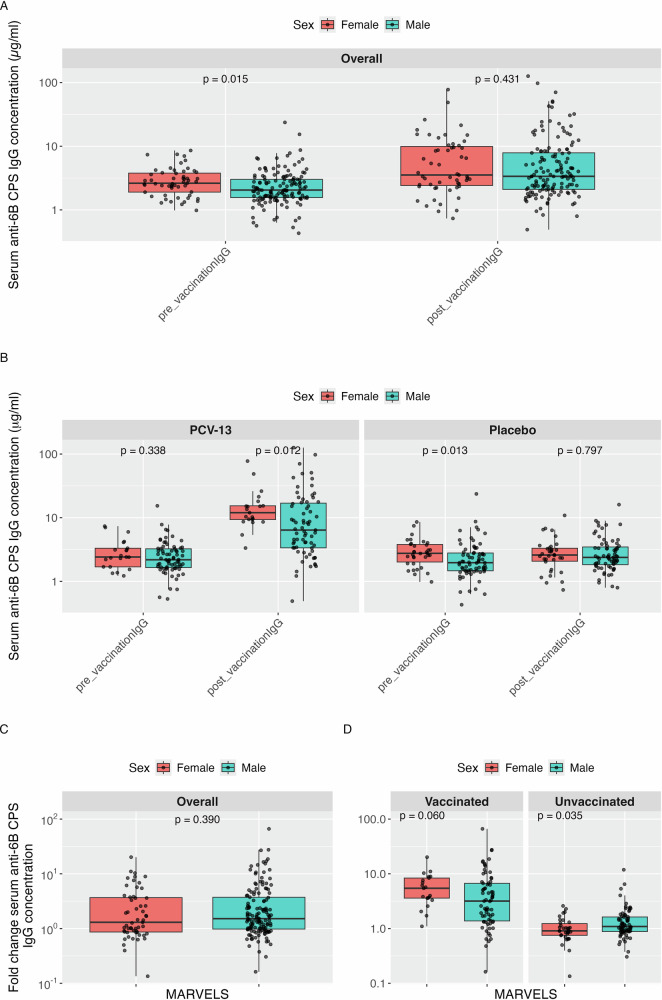
MARVELS CPS IgG antibody concentration at pre-vaccination and post-vaccination, and CPS IgG fold change pre-post-vaccination. Serum CPS IgG concentration changes and fold changes pre- and post-vaccination in the MARVELS cohort, with data stratified by sex and vaccination status. Data are presented as box plots displaying the median, interquartile range (IQR). There were 7 observations with missing CPS IgG measurements at both visits. **A** Overall CPS IgG concentrations and stratified by sex at pre-vaccination and post-vaccination. **B** CPS IgG concentrations stratified by sex and vaccination status (PCV-13 vs. Saline) at pre-vaccination and post-vaccination. **C** Overall fold changes in CPS IgG concentrations post-vaccination compared to pre-vaccination, shown overall by sex. **D** Fold changes in CPS IgG concentrations post-vaccination compared to pre-vaccination, stratified by sex and further vaccination status.

In the Malawi population, a statistically significant difference in the baseline serum anti- CPS IgG levels was observed between males and females, with lower IgG titres in males (median = 2.05 µg/ml in males and median = 2.62 µg/ml in females, *p* = 0.015) Table 5. However, this baseline difference was reduced and no longer statistically significant post-vaccination (median = 3.37 µg/ml in males, 3.53 µg/ml in females; *p* = 0.431; Fig. 1). When stratified by vaccination status, the vaccinated group showed no statistically significant difference in baseline anti CPS-IgG levels between females (median = 2.42 µg/ml) and males (median = 2.19 µg/ml, *p* = 0.338), but female participants had significantly higher anti-CPS IgG titres post-vaccination than male vaccinees (median = 12.00 vs 6.40 µg/ml; *p* = 0.012). The unvaccinated group had median IgG value of 2.76 µg/ml for females vs 1.97 µg/ml for males at baseline *p* = 0.013 while at 4 weeks post-vaccination the values did not differ by sex; 2.60 µg/ml for females and 2.38 µg/ml for males *p* = 0.797. When comparing IgG fold change from baseline to post-vaccination between males and females, in the vaccinated group, females showed a higher fold change than males (5.45 vs 3.18, *p* = 0.060), the unvaccinated group had median IgG fold change value of 0.91 in females and 1.09 in males *p* = 0.035 (Fig. 1).

**Table 5:** Summary of IgG levels (µg/mL) against serotype 6B pre- and post-vaccination

Setting	*N*	Pre-vaccination	*p*-value	Post-vaccination	*p*-value	Fold change	*p*-value
**MARVELS**							
Overall	197	2.30 (1.6–3.25)		3.37 (2.22–8.6)		1.46 (0.91–3.74)	
Female	55	2.62 (1.9–3.78)	0.015	3.53 (2.42–9.93)	0.431	1.30 (0.86–3.68)	0.390
Male	142	2.05 (1.56–3.02)		3.37 (2.1–7.88)		1.52 (0.98–3.73)	
Female: Vaccinated	21	2.42 (1.69–3.32)	0.338	12.00 (9.37–15.38)	0.012	5.45 (3.6–8.37)	0.060
Male: Vaccinated	71	2.19 (1.65–3.25)		6.40 (3.36-16.94)		3.18 (1.38–6.70)	
Female: Unvaccinated	34	2.76 (2.03–3.80)	0.013	2.60 (2.07–3.32)	0.797	0.91 (0.75–1.2)	0.035
Male: Unvaccinated	71	1.97 (1.47–2.79)		2.38 (1.83-3.48)		1.09 (0.88–1.63)	
**EHPC**							
Overall	92	0.72 (0.54–1.12)		1.47 (0.69–3.49)		1.17 (0.92–4.97)	
Female	56	0.80 (0.57–1.38)	0.089	1.57 (0.77–3.12)	0.445	1.16 (0.88–5.28)	0.971
Male	36	0.66 (0.4–0.96)		1.29 (0.59–3.51)		1.17 (0.95–4.29)	
Female: Vaccinated	27	0.70 (0.57–1.16)	0.707	2.98 (1.76–7.28)	0.825	5.50 (2.33–6.79)	0.860
Male: Vaccinated	19	0.71 (0.58–0.91)		3.48 (1.62–6.5)		4.08 (2.28–7.67)	
Female: Unvaccinated	29	0.89 (0.59–1.62)	0.092	0.84 (0.48–1.56)	0.055	0.92 (0.82–1.04)	0.443
Male: Unvaccinated	17	0.59 (0.3–1.00)		0.57 (0.28–0.91)		0.94 (0.76–1.04)	
**Pooled IgG**							
Overall	289	1.80 (0.99−2.75)		2.93 (1.59−6.84)		1.40 (0.91−4.00)	
Female	111	1.64 (0.80−2.68)	0.287	2.56 (1.20−7.29)	0.155	1.20 (0.87−4.18)	0.403
Male	178	1.83 (1.20−2.76)		3.25 (1.79−6.80)		1.47 (0.96−3.91)	
Female: Vaccinated	48	1.45 (0.68−2.45)	0.073	8.52 (2.59−15.01)	0.706	5.47 (3.00−7.05)	0.053
Male: Vaccinated	90	1.88 (1.21−3.00)		5.65 (3.05−13.62)		3.30 (1.51−6.71)	
Female: Unvaccinated	63	1.69 (0.94−2.90)	0.839	1.71 (0.85−2.81)	0.141	0.91 (0.79−1.06)	0.026
Male: Unvaccinated	88	1.72 (1.21−2.63)		2.10 (1.39−3.27)		1.03 (0.87−1.45)	

This table presents median IgG concentrations (in µg/mL) and interquartile ranges (IQR: 25th–75th percentile) measured before and after vaccination with PCV-13. Fold change is calculated as the ratio of post- to pre-vaccination IgG levels. Results are stratified by study setting (EHPC and MARVELS), sex, and vaccination status. We have included pooled IgG distributions from the MARVELS and EHPC IgG data. *N* is the total sample size in each group. *P*-values are from Wilcoxon rank-sum tests comparing IgG levels between groups.

**Fig. 2:**
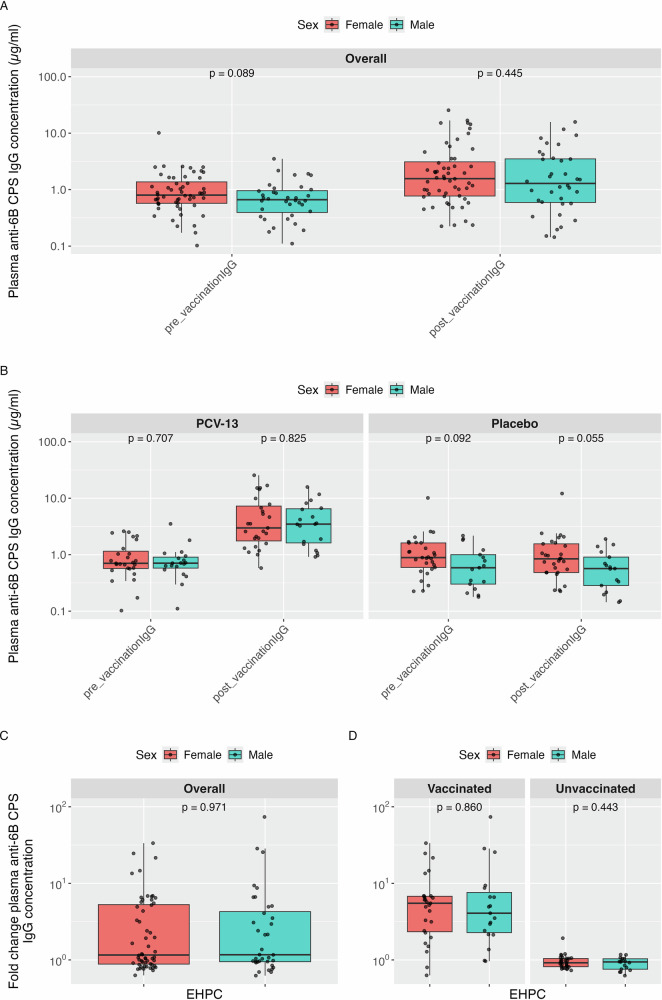
EHPC plasma IgG antibody concentration pre-vaccination and post-vaccination and IgG fold change pre-post-vaccination. CPS IgG concentration changes and fold changes pre- and post-vaccination in the EHPC cohort, with data stratified by sex and vaccination status. Data are presented as box plots displaying the median, interquartile range (IQR). There were 4 observations with missing CPS IgG measurements at both visits. **A** Overall CPS IgG concentrations and stratified by sex at pre-vaccination and post-vaccination. **B** CPS IgG concentrations stratified by sex and vaccination status (PCV-13 vs. Hepatitis A (Avaxim)) at pre-vaccination and post-vaccination. **C** Overall fold changes in CPS IgG concentrations post-vaccination compared to pre-vaccination, shown overall by sex. **D** Fold changes in CPS IgG concentrations post-vaccination compared to pre-vaccination, stratified by sex and further vaccination status.

In the UK population, no statistically significant differences in anti-CPS IgG values between males and females were observed pre- and post-vaccination, while the unvaccinated females had significantly higher IgG values 0.84 µg/ml than males 0.57 µg/ml *p* = 0.055 post-vaccination Table 5. The baseline IgG levels were slightly higher in females (median = 0.80 µg/ml than in males and median = 0.66 µg/ml in females, *p* = 0.089) and at post-vaccination, anti-CPS IgG median concentration was 1.57 µg/ml in females vs 1.29 µg/ml in males (*p* = 0.445; Fig. 2). When stratified by vaccination status, the vaccinated group showed baseline median IgG value of 0.70 µg/ml in females and 0.71 µg/ml in males; *p* = 0.707, and 2.98 µg/ml in females vs 3.48 µg/ml in males at post vaccination *p* = 0.825. The unvaccinated group had baseline IgG values of 0.89 µg/ml in females vs 0.59 µg/ml in males *p* = 0.092. At post vaccination, females had median IgG value of 0.84 µg/ml vs 0.57 µg/ml in males *p* = 0.055. The median overall fold change was 1.16 in females and 1.17 for males *p* = 0.971 with 5.50 in females vs 4.08 for males (*p* = 0.860) and 0.92 for females vs -0.94 for males (*p* = 0.443) in the vaccinated and unvaccinated groups respectively (Fig. 2).

**Fig. 3:**
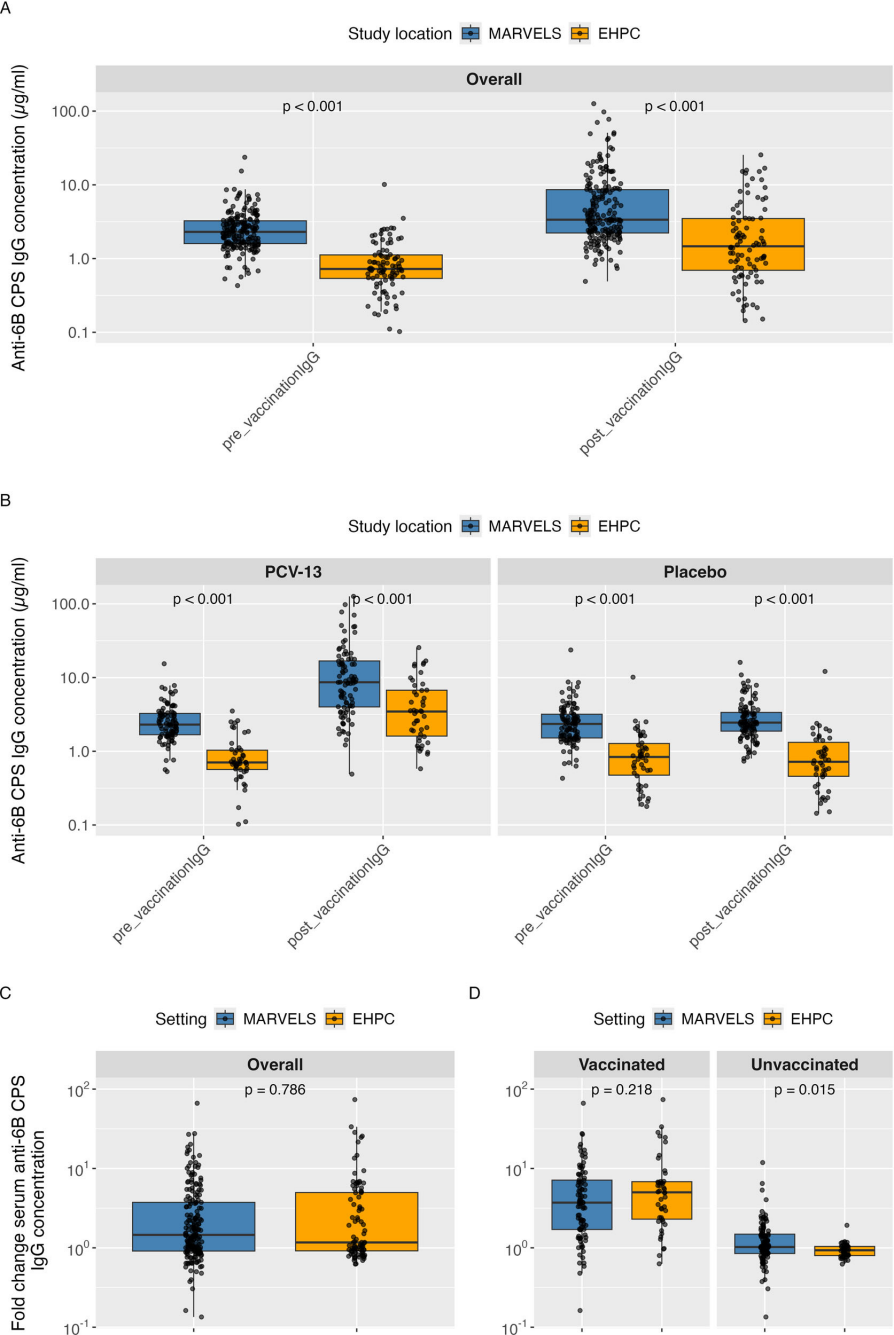
CPS IgG antibody Concentration between MARVELS and EHPC at pre- and post-vaccination. Comparison of CPS IgG concentrations between the MARVELS and EHPC cohorts at pre- and post-vaccination. **A** Overall, MARVELS and EHPC CPS IgG concentrations at pre-vaccination and post-vaccination. **B** MARVELS and EHPC CPS IgG concentrations stratified by vaccination status at pre-vaccination and post-vaccination **C** Overall MARVELS and EHPC fold changes in CPS IgG concentrations post-vaccination compared to pre-vaccination stratified by sex. **D** MARVELS and EHPC fold changes in CPS IgG concentrations post-vaccination compared to pre-vaccination, stratified by sex and vaccination status.

Across both studies, there was a striking difference in baseline anti-capsular IgG with the Malawi population showing a higher IgG levels at baseline (median = 2.30 µg/ml vs 0.72 µg/ml *p* < 0.001) and post-vaccination (median = 3.37 µg/ml vs 1.47 µg/ml; *p* < 0.001; Fig. 3). An increase in anti-CPS IgG levels was observed following PCV-13 vaccination in both sites as shown in Table 5. In the MARVELS study, IgG levels increased from a baseline median of 2.30 µg/ml to 3.37 µg/ml post-vaccination (*p* < 0.001). In the EHPC study, IgG levels increased from a baseline median of 0.72 µg/ml to 1.47 µg/ml post-vaccination (*p* < 0.001) Fig. 3.

**Fig. 4:**
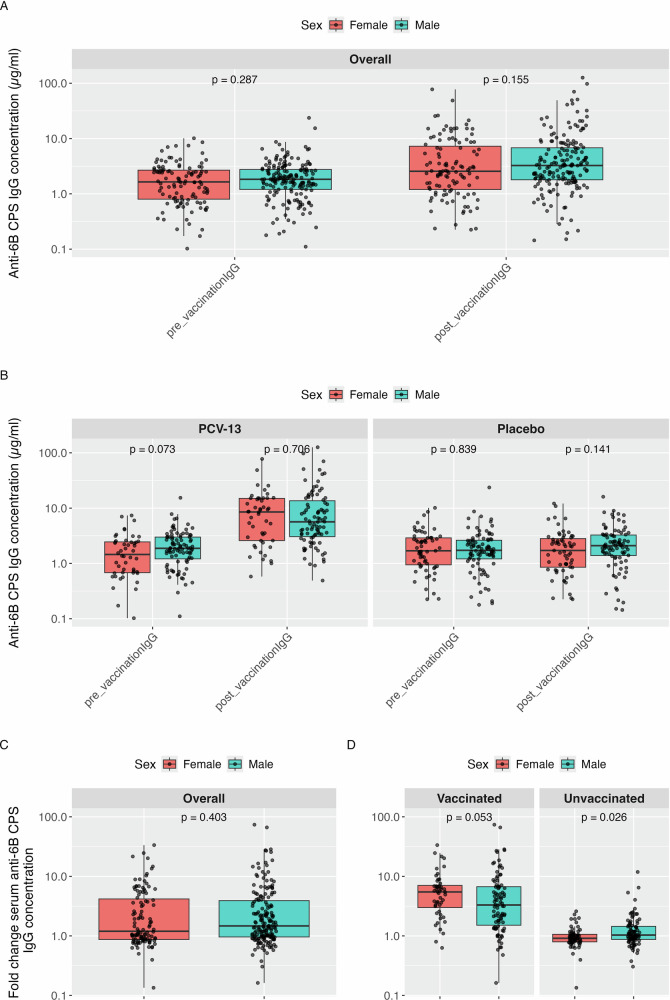
Pooled CPS IgG antibody Concentration from MARVELS and EHPC at pre- and post-vaccination. Pooled CPS IgG concentrations from the MARVELS and EHPC cohorts at pre- and post-vaccination. (**A)** Overall, pooled CPS IgG concentrations at pre-vaccination and post-vaccination. (**B)** Pooled CPS IgG concentrations stratified by vaccination status at pre-vaccination and post-vaccination (**C**) Overall Pooled fold changes in CPS IgG concentrations post-vaccination compared to pre-vaccination, stratified by sex. **(D)** Overall Pooled fold changes in CPS IgG concentrations post-vaccination compared to pre-vaccination, stratified by sex vaccination status.

A pooled, post-hoc analysis of CPS-specific IgG responses from both MARVELS and EHPC included 289 participants Table 5. Overall, median IgG concentrations increased from 1.80 µg/mL (IQR: 0.99–2.75) at baseline to 2.93 µg/mL (IQR: 1.59–6.84) post-vaccination. When stratified by sex, females had slightly lower median pre-vaccination titres (1.64 µg/mL, IQR: 0.80–2.68) compared to males (1.83 µg/mL, IQR: 1.20–2.76), though this was not statistically significant (*p* = 0.287). Post-vaccination titres remained slightly lower in females (2.56 µg/mL, IQR: 1.20–7.29) compared to males (3.25 µg/mL, IQR: 1.79–6.80), *p* = 0.155. Among vaccinated participants, the median fold change in IgG titres was higher in females (5.47, IQR: 3.00–7.05) than in males (3.30, IQR: 1.51–6.71), with a marginally non-significant *p*-value of 0.053. In contrast, unvaccinated participants showed minimal changes, with females exhibiting a small but statistically significant reduction in fold change (0.91, IQR: 0.79–1.06; *p* = 0.026), while males showed no significant change (1.03, IQR: 0.87–1.45) Fig. 4.

## Discussion

We performed an analysis of pooled data from the MARVELS programme in Malawi and the EHPC programme in Liverpool, UK. By leveraging the similarities in the study designs, challenge agents, standard operating procedures for sample processing and IgG ELISA, the pooled analysis allowed us to examine sex differences in pneumococcal carriage rates, PCV-13 vaccine efficacy, and immune response. To our knowledge, this pooled analysis represents the first to combine data across two studies from two different pneumococcal controlled human challenge programmes (MARVELS and EHPC) to jointly assess PCV-13 vaccine protection against pneumococcal carriage using a principled statistical framework. While the studies have each previously reported the vaccine efficacy, our approach demonstrates the feasibility of integrating CHIM data from distinct geographical and programmatic settings. By leveraging data from multiple studies, the analysis improves statistical power and enables more precise estimation of vaccine efficacy. This work lays the foundation for future methodological harmonisation between CHIM cohorts globally. It highlights the potential of integrative data analysis techniques in vaccine evaluation, particularly where logistical or ethical considerations may limit lager sample sizes.

Overall, the PCV-13 vaccine significantly reduced the risk of Spn6B carriage in both study populations. The pooled analysis associated with PCV13 vaccination suggests a 76% (95% CI; 51–90%) reduction in the likelihood of experimental pneumococcal carriage highlighting the vaccine’s efficacy in preventing mucosal colonisation, a prerequisite for community transmission. This protective effect was consistently observed in both MARVELS (72% reduction; Table 2, log-binomial model RR 0.28) and EHPC (86% reduction; Table 3, log-binomial model RR 0.14) populations. Our analysis showed a consistent protective effect of PCV-13, confirming previous findings that PCVs provide effective protection against carriage^15,23–26^—a critical factor in reducing bacterial transmission and invasive disease, particularly in sub-Saharan Africa. We also observed a significant reduction in carriage in the 20,000 CFU group compared to the 160,000 CFU group, consistent with prior observations that lower inoculation dose results in lower carriage rates^27^. A sensitivity analysis conducted to assess the potential influence of inoculation dose on the observed vaccine effect showed an almost identical reduction in carriage risk, indicating that the vaccine effect is unlikely to be influenced by dose level in our study.

While no statistically significant association was observed between sex and pneumococcal carriage rates, female participants had slightly higher carriage rates than males across both studies. Although the pooled analysis increased the sample size, it was not sufficiently powered to detect small differences by sex. However, the direction of the observed effect aligns with our previous work suggesting men may be less likely to carry pneumococcus^5^. Carriage has been shown to confer an immunological boost which may provide some protection against IPD. Additionally, experimental carriage rates were higher in the EHPC study compared to MARVELS. These differences may reflect variations in environmental or behavioural factors unique to each setting, influencing pneumococcal transmission dynamics. Technical factors such as sampling timepoints, swab techniques or laboratory methods could contribute to the observed variations in carriage. However, in mitigation, the protocols used were near-identical, laboratory methods similar or identical, and the investigator teams had overlapping personnel so that these differences are expected to be small.

Previous studies have documented differences between men and women in their susceptibility to pathogens, carriage rates, immune responses to illnesses, and vaccination outcomes^4,6,28–31^. These differences extend to disease severity and treatment responses. The analysis of IgG responses revealed some sex-based differences in immune responses following vaccination. In the MARVELS study, vaccinated females had significantly higher IgG levels compared to males, suggesting that females may generate stronger humoral immune responses compared to males. This observation aligns with evidence from vaccine studies indicating that oestrogen may enhance antibody production, contributing to stronger immune responses in females^32–34^. Conversely, in the unvaccinated group, males exhibited higher IgG levels than females. However, these sex differences were not replicated in the EHPC study, suggesting that other factors may influence immune responses across different study settings. The findings between MARVELS and EHPC were not consistent and may be confounded by the sample type, standards, population differences or other unmeasured factors and the observed differences may not translate into meaningful variation in protection, and that this study was not designed to assess correlates of protection. However, it remains unclear whether these differences result in reduced carriage rates or disease burden^6,18,28,29^. Sex-based differences in immune responses may be driven by hormonal and genetic factors influencing immunity^35^, though the exact mechanisms remain unknown.

The complex interplay of social, behavioural, and biological factors likely contributes to the observed sex differences. Previous studies have shown that younger females often exhibit stronger inflammatory, antibody, and cell-mediated immune responses to vaccines than males^23^. In our pooled analysis, vaccinated females exhibited higher post-vaccination IgG concentrations and fold changes compared to males, which is consistent with these earlier findings. The analysis was not powered for this effect, but the direction of the effect aligns with existing evidence, supporting the biological plausibility of sex-based immune variation. These findings highlight the need for further investigation into sex-differentiated vaccine responses, especially in settings with differing exposure histories and background immunity. Such insights could inform the development of tailored vaccine strategies that address sex-specific differences in immunity and disease risk

The baseline IgG antibody levels in both cohorts exceeded the published threshold of 0.5 µg/ml for protection against serotype 6B carriage^36^. However, MARVELS participants had significantly higher baseline IgG levels compared to EHPC participants. Higher baseline IgG titres observed among participants in Malawi is likely a result of more frequent natural boosting through repeated pneumococcal exposure in this high- exposure setting^16^. While higher baseline titres may contribute to some level of protection, it is also possible that in high-transmission settings, naturally boosted immunity may not fully compensate for the burden of pneumococcal disease, particularly in vulnerable groups such as infants or immunocompromised individuals, underscoring the continued importance of vaccine strategies tailored to local populations. In this study, the experimental serotype 6B carriage rate was 20% in MARVELS and 29% in the EHPC during these trials. While environmental and behavioural factors may contribute to this variation, other explanations include the differences in baseline immunity influenced by prior natural carriage or differences in host immunity due to early-life exposure. Previous studies suggest that infants from LMICs have significantly higher maternal-derived IgG levels compared to those from high-income countries (HICs)^36^. Additionally, the protective thresholds for infants in LMICs were reported to be 2.15 times higher than for infants in HICs^36^. These findings are consistent with the higher force of infection observed in LMICs compared to HICs^37^, and with our dose-dependent rates of experimental human pneumococcal carriage. Increased exposure and natural carriage may contribute to the observed differences in baseline IgG levels between MARVELS and EHPC participants. Historical infant vaccination programmes may also have an influence on adult immunity through indirect effects on herd immunity and transmission dynamics^20,38^.

In this analysis, neither the MARVELS nor EHPC studies were powered to investigate sex-based differences in vaccine efficacy. While combining data from both programmes improved statistical power, we did not observe significant differences in vaccine efficacy between sexes. However, the results form a foundation for future research and underscore the need for larger, adequately powered studies to better understand sex differences. Future investigations should explore inflammation, genetic, social and environmental factors contributing to these variations. In addition, there was a notable imbalance in the sex distribution between the study populations, with 72% of participants in the Malawi (MARVELS) study being male, compared to 39% in the UK (EHPC) study. This discrepancy may have influenced the sex-stratified analyses and should be considered when interpreting the results. Given the exploratory and hypothesis-generating nature of our analyses, multiple comparisons were not adjusted for. We acknowledge this may increase the likelihood of a type I error. There was also a substantial epidemiological and contextual heterogeneity between Malawi and the UK in this pooled analysis. These include differences in population structure, nutritional status, force of infection, prior pneumococcal exposure, and health system factors, all of which may influence both baseline IgG levels and experimental carriage dynamics. While the biological mechanism of vaccine-induced protection is likely consistent, these contextual factors may explain some of the variability observed. The setting effect in our pooled model was near statistically significant, suggesting an independent influence of study site on carriage outcomes. We accounted for this by including study setting as a covariate in all pooled models. We also assumed the biological mechanism of the vaccination protection to be the same in both settings despite heterogeneity in overall carriage levels. Additionally, there were differences in the reference standards used for quantifying serotype 6B-specific IgG between the MARVELS and EHPC datasets. The use of different IgG reference standards (007sp for MARVELS and 89SF for EHPC) is noteworthy but is unlikely an important methodological consideration for interpreting and comparing absolute titre values between the two cohorts. Although previous studies and internal data from the EHPC programme have demonstrated good concordance between these standards^39^, subtle differences in assay calibration and reference range may impact the comparability of absolute IgG concentrations. By acknowledging these methodological differences, we aimed to avoid introducing bias or misinterpretation in the pooled analysis, particularly given the different sample types (serum vs plasma) used across studies even though internally validated. Future work should incorporate harmonised reference materials and standardised assay protocols across multicentre CHIM studies.

This pooled analysis of MARVELS and EHPC programmes highlights the overall effectiveness of PCV-13 in reducing pneumococcal carriage across distinct settings and provides a more precise estimate than that obtained from either study alone. No significant sex differences in vaccine efficacy or immune responses were observed, but the limited power to detect such interactions even for the pooled analysis warrants cautious interpretation. We observed setting-related differences in carriage outcomes and immune responses, highlighting the importance of accounting for study context when interpreting CHIM data. Importantly, our findings demonstrate the feasibility, value and limitations of pooling data across CHIM studies. This creates a platform for future pooled analyses to enhance statistical power, explore sub-group effects and strengthen the evidence base for pneumococcal vaccine strategies.

## Methods

### Study design and setting

This study involved a secondary data analysis of data from previously published CHIM studies; MARVELS study conducted in Blantyre Malawi and EHPC conducted in Liverpool UK. The data for this analysis were pooled from separate trials conducted at different times (2013 for EHPC and 2020 for MARVELS). Therefore, this pooled, secondary analysis was post-hoc as it was not pre-specified as part of either trial’s protocol or statistical analysis plan. Both studies followed harmonised protocols and were approved by research ethics committees. The MARVELS received ethical approvals from Malawi by the National Health Sciences Research Committee (16/07/2519) and the Pharmacy Medicines and Regulatory Authority (PMRA/CRTC/III/10062020121) and in the UK by the Liverpool School of Tropical Medicine^15^. Whereas the EHPC received ethical approval by the National Health Service Research and Ethics Committee (12/NW/0873 Liverpool)^14^. All participants gave written informed consent which included re-use of data. The analysis was meant to be hypothesis generating, while trying to increase power for detecting sex effects by pooling across both trials and the sex-stratified findings are exploratory and should be interpreted with caution.

The Malawi study (MARVELS trial) was registered with the Pan African Clinical Trial Registry (PACTR202008503507113) on 03 August 2020. The UK study (EHPC trial) was registered in the ISRCTN registry (ISRCTN45340436) on 18 November 2013. Details of the study designs and settings have been previously described for MARVELS^15^ and EHPC^14^. The MARVELS and EHPC studies were double-blinded (research staff and participants were blinded to the vaccination allocation), parallel-arm, randomised controlled trials investigating the efficacy of PCV-13. To note that the outcomes, including carriage status, were assessed within each study in accordance with the double-blind design to avoid bias. As the data had been unblinded at data lock for each study, for the pooled analysis, the investigators were not blinded. In MARVELS, the allocation ratio was 1:1 for PCV-13 and saline, while in EHPC, it was 1:1 for PCV-13 and Hepatitis A (Avaxim) vaccine. Randomisation sequencies were computer generated, and the randomisation schedule was produced by an independent statistician, using block randomisation with random block sizes of 6, 8, and 10 for MARVELS and block sizes of 10 for EHPC. Both studies focused on experimental pneumococcal carriage of *Streptococcus pneumoniae* serotype 6B (Spn6B). The studies evaluated other serotypes including vaccine type and non-vaccine type serotypes but for this analysis we have focused on experimental serotype 6B only.

Both studies involved a vaccination visit, during which participants in the intervention arm received the PCV-13 vaccine followed by an inoculation visit 28 days later, where participants were experimentally inoculated with pneumococcal serotype 6B. Experimental pneumococcal carriage was assessed by classical microbiological culture of nasal washes at days 2, 7, and 14 post inoculation for MARVELS, and days 2, 7, 14, and 21 post inoculation for EHPC^14,15,40,41^. All carriage results reported in this paper are based on the post-inoculation measurements, which also corresponds to post-vaccination assessments.

### Inclusion and exclusion criteria

Health adult volunteers aged 18-40 (MARVELS), 18-50 (EHPC) were enroled in the study. Participants were eligible for enrolment if they gave written informed consent, were HIV negative, no prior pneumococcal vaccination. Exclusion criteria included current respiratory infection, pregnancy or breast feeding, chronic illness, close physical contact with at risk individuals as previously described in the study protocols^40,41^. Despite differing national immunisation schedules, all participants included in this pooled analysis were confirmed to be PCV-13-naïve at enrolment. Further protocol details have been outlined in the supplementary Table 3.

### Data Integration and harmonisation

A simplified approach was implemented for pooling data from both settings. Data were integrated into a single database, and we aligned and standardised data variables across datasets to ensure consistency. The variables included age, sex, vaccination status (PCV-13 or control), carriage outcomes, and IgG concentrations. Units for antibody concentrations (µg/mL) and time points for sample collection (baseline, days 2, 7, and 14 post-inoculation) were standardised to maintain uniformity across the datasets. No missing data were present for demographic variables and outcome variable, as participants with incomplete data were replaced by design. For analyses involving IgG concentrations, we used a complete-case approach. For this analysis, pneumococcal carriage status of the participants was defined as carriage at any of the study visits on days 2, 7, and 14 post-inoculation for both studies. This decision was made as part of data harmonisation and prior to accessing the outcomes for the pooled analysis. It is important to note that, despite extending the observation time to day 21, in the EHPC study, no additional carriers were identified at this additional time point.

### Study baseline data and vaccination

Baseline nasal and serum screening samples were collected to exclude natural carriage of Spn6B and to establish baseline burdens of natural carriage for other VTs and non-vaccine types (NVTs) of pneumococcus. Participants were allocated to two study arms and were vaccinated with either PCV-13 or control/placebo (control was saline placebo for MARVELS and Hepatitis A (Avaxim) for EHPC).

### Inoculation

At 28 days post-vaccination, participants were inoculated with pneumococcus serotype-6B. In MARVELS, escalating doses of 20,000 CFU/100 ml (40 participants), 80,000 CFU/100 ml (74 participants) or 160,000CFU/100 ml (90 participants) per naris were administered. In EHPC, participants received a single dose of 80,000CFU/100 ml (96 participants) per naris^14,15^.

### Pneumococcal anti-capsular polysaccharide antibody ELISA

We measured anti-serotype 6B Capsular Polysaccharide Immunoglobulin G antibodies to pneumococcal capsular polysaccharide using the World Health Organization’s ELISA protocol, as previously described^39^. For this study, ELISA plates were coated with 5 µg/ml of serotype-6B or 15B capsular polysaccharide (control) and incubated at 2–8 °C overnight. Samples were serially diluted, added to the plates in triplicate, and incubated at room temperature. After washing, goat anti-human IgG- alkaline phosphatase (Southern Biotech) was added, and optical densities were measured at 405 nm after substrate incubation. Antibody concentrations were calculated using a four-parameter logistic curve in the MyAssays platform as previously described^18,42^. The serum samples were analysed using reference standards 007sp (National Institute for Biological Standards and Control, UK) for MARVELS and 89SF EHPC as 007sp was not available during the study implementation. To standardise the units of measurements we converted the EHPC IgG values from nanograms per millilitre (ng/ml) to micrograms per millilitre (µg/ml) by dividing the IgG values by 1000.

### Statistical analysis

Descriptive statistics, including frequencies and percentages, were calculated to summarise demographic characteristics, vaccination status, and sex (self-reported by participant at the time of enrolment) in both the MARVELS and EHPC studies and we calculated exact 95% binomial confidence intervals for all proportions. Pneumococcal carriage proportions between males and females were compared using Fisher’s exact test as the sample sizes made this test computationally feasible to calculate and as it does not rely on a normal approximation (as inherent to the chi-squared test).

Log-binomial models were used to estimate the relative risk of experimental pneumococcal carriage associated with vaccination and sex. Separate models were fitted for each programme (MARVELS, EHPC). For each study-specific model, the outcome variable was experimental pneumococcal carriage (yes/no) and independent variables included study arm (vaccination status; PCV-13 vs Control) and sex (male vs female), and an interaction term for vaccination status and sex. For MARVELS, inoculation dose (20,000, 80,000, 160,000 CFU) was included as an additional independent variable.

In the pooled analysis, we fitted a log-binomial model with experimental carriage as the binary response variable. Independent variables included vaccination status, sex, study setting, and an interaction term for vaccination status and sex. The reference categories in these models were: Control for vaccine type, male for sex, 160,000 CFU for dose in the MARVELS specific model (80,000 CFU for the pooled sensitivity analysis model) and MARVELS for study setting. We accounted for study setting by including it as a fixed effect in the pooled analysis model. We did not adjust for multiple comparisons, as the analyses were exploratory and each comparison was for a separate endpoint.

We compared CPS-specific IgG concentrations between males and females pre-vaccination (baseline) and at post-vaccination (4 weeks post-vaccination) within each setting. IgG fold change defined as the ratio of the post-vaccination IgG and the pre-vaccination IgG, i.e. post-vaccination IgG/ pre-vaccination IgG, was calculated and compared between sexes and vaccination groups. The fold change quantifies the magnitude of change in the antibody levels relative to the baseline (pre-vaccination) values between groups. The IgG fold changes were compared between sexes using the Wilcoxon rank-sum test. IgG levels at specific time points (pre-vaccination, post-vaccination) were compared between sexes using Wilcoxon signed-rank test. Only participants with IgG measurements at both visits were included in the fold change analysis. We conducted a post-hoc pooled IgG analysis using IgG data from MARVELS (serum, 007sp standard) and EHPC (plasma, 89SF standard) participants. Internal validation in EHPC confirmed comparability between serum and plasma, supporting the pooled analysis. All statistical analyses were conducted using R version 4.3.1 software with a significance level of 0.05. The following R packages were used: logbin^43^ for fitting log-binomial models to estimate relative risks, ggpubr^44^, ggplot2^45^, patchwork^46^, and gridExtra^47^ for data visualisation and figure arrangement, table1^48^ for generating descriptive summary tables, dplyr^49^ and tidyr^50^ for data manipulation, lubridate^51^ for date handling, and readr^52^ for data import.

### Sensitivity analysis

Given the difference in inoculation doses between the MARVELS (20,000, 80,000, and 160,000 CFU) and EHPC (80,000 CFU) studies, we conducted a sensitivity analysis to assess the impact of dose on experimental carriage by adjusting our pooled model with dose level as a covariate.

## Supplementary information

Supplementary information

## Data Availability

The dataset generated and/or analysed during this study are publicly available on Figshare repository and can be accessed at: 10.6084/m9.figshare.28261703.
