# Supplementary material for: Pooled analysis of PCV13 efficacy from controlled human infection trials in Malawi and the UK
Source: NPJ Vaccines. 2026 Feb 26;11:101. doi: 10.1038/s41541-026-01381-4 (PMC13176328; doi:10.1038/s41541-026-01381-4)
Supplement: Supplementary file 1 — Supplementary information [file 41541_2026_1381_MOESM1_ESM.pdf]

Supplementary Table 1: Combined log-binomial model summary for Malawi and EHPC population adjusted for dose

	Estimate	95% CI	SE	Z statistic	p-value
(Intercept)	0.333	0.215-0.516	0.223	-4.926	0.000
PCV-13 vaccine	0.245	0.113-0.528	0.393	-3.586	**0.000**
Dose: CFU 20,000	0.244	0.061-0.978	0.708	-1.992	**0.046**
Dose: CFU 160,000	0.998	0.584-1.705	0.273	-0.008	0.993
Female	1.086	0.703-1.679	0.222	0.372	0.710
EHPC	1.288	0.785-2.115	0.253	1.002	0.316
PCV-13 vaccine:Female	1.547	0.546-4.384	0.531	0.821	0.412

Supplementary Table 1 legend: Log-binomial regression model results evaluating the effect of inoculation dose, vaccination status, sex, and study site on the relative risk (RR) of experimental pneumococcal carriage (serotype 6B). The model includes dose levels (reference: 80,000 CFU), study site (reference: MARVELS), sex (female vs. male), and an interaction between PCV-13 vaccine and sex. Estimates are presented as relative risk (RR) values, 95% confidence interval (95% CI) with standard error (SE), Z statistic, and p-value. RR < 1 indicates reduced risk of carriage. The total sample size for this model is N = 300.

Supplementary Table 2: Carriage Rates and 95% Confidence Intervals by Inoculation Dose and Study Setting

Setting	dose	N	Carriage_Positive	Carriage_Rate (95% CI)
EHPC	80000 cfu	96	28	29.2% (21.0–38.9)
MARVELS	20000 cfu	40	2	5.0% (1.4–16.5)
MARVELS	80000 cfu	74	18	24.3% (16.0-35.2)
MARVELS	160000 cfu	90	20	22.2% (14.9–31.8)

Supplementary Table 2 legend: Descriptive summary of experimental carriage rates and the 95% binomial confidence interval (95% CI) by study setting and inoculation dose group. Carriage was defined as positive culture for serotype 6B at any follow-up timepoint (days 2, 7, or 14 post-inoculation). **Note:** EHPC used a single dose (80,000 CFU), while MARVELS had three escalating doses (20,000 CFU, 80,000 CFU and 160,000 CFU).

Supplementary Table 3: Summary of the study procedures from MARVELS and EHPC

**Category**	**MARVELS**	**EHPC**
**Main objective**	Determine efficacy of PCV-13 vaccination against experimental pneumococcal carriage	Determine effect of PCV-13 vaccination on experimental pneumococcal colonisation
**Strain**	S.pneumoniae serotype 6b BHN418	*S. pneumoniae* serotype 6B BHN418
**Primary endpoint**	Detection of 6B pneumococcus in nasal wash by classical microbiology	Detection of 6B pneumococcus in nasal wash by classical microbiology
**Secondary endpoint**	Carriage density and duration, immune response parameters	Carriage density and duration,
**Detection of carriage**	Nasal wash on days 2, 7, 14 post-inoculation with culture and PCR confirmation	Nasal wash on days 2, 7, 14, 21 post-inoculation with Culture
**Termination of Carriage**	All carriers receive 3-day amoxicillin course	All carriers receive 3-day amoxicillin course
**Study design**	Double-blinded, parallel-arm randomised controlled trial	Double-blinded randomized controlled trial
**Study setting**	Queen Elizabeth Central Hospital & MLW Labs, Blantyre, Malawi	LSTM & Royal Liverpool University Hospital, UK
**Recruitment target**	200 participants	100 participants
**Recruitment**	12 months	9 months
**Screening and recruitment**	Healthy adults aged 18–40; community advertising and informed consent process	Healthy adults aged 18–50, poster advertisements and on the University website
**Randomization**	Block randomisation (1:1) to PCV-13 or .0.9% saline (control); random block sizes of 6, 8, 10	Block randomisation (1:1!) to PCV-13 or hepatitis A vaccine (control) in blocks of 10.
**Post vaccination samples to confirm carriage**	Collected before inoculation and post-inoculation (blood, nasal wash, throat and saliva)	Collected pre-vaccination, post-vaccination, and post-inoculation<br>(Blood, nasal wash, nasal swabs, throat swabs)
**Dose**	Escalating: 20,000 (n=40) → 80,000 (n=140) → 160,000 (n=20)	Fixed 80,000 CFU
**Inoculation**	S. pneumoniae 6B inoculation via nasal inoculation inside each naris in three dose groups: 20,000, 80,000, 160,000 CFU	S. pneumoniae 6B nasal inoculation with 80,000 CFU inside each naris
**Immunology response measurements**	Serum IgG, saliva, nasal wash IgG, cellular responses, cytokines, transcriptomics, microbiome	Serum IgG, nasal wash IgG, cytokines, nasal wash, microbiome
**Antibody titres**	Measured using ELISA	Measured using ELISA
**Inclusion criteria**	• Age 18–40<br>• Chichewa/English fluency<br>• Healthy adults<br>• not in contact with vulnerable individuals (children <5 years, immunosuppressed adults, elderly, chronic ill health)	• Age 18–50<br>• English-speaking<br>• Healthy<br>• Able to text<br>• No contact with at-risk individuals (children <5 years, immunosuppressed adults, elderly, chronic ill health)

**Exclusion criteria**	• Unable to give consent<br>• Previous pneumococcal vaccination<br>• Close physical contact with at-risk groups<br>• Allegic to penicilin/amoxicillin<br>• Acute illness<br>• Chronic illness<br>• On immunosuppressive medication<br>• Pregnancy<br>• Involvement in another clinical trial<br>• History of alcohol or drug abuse<br>• Positive for spn 6B.<br>• SARS-CoV-2, HIV+	• Unable to give fully informed consent.<br>• Previous pneumococcal vaccination<br>• Close contact with at-risk groups<br>• Penicillin/ amoxicillin allergy,<br>• Asthma, other respiratory disease,<br>• pregnancy<br>• Current involvement in another clinical trial unless observational or in non-interventional phase.<br>• Smoker or history >10 pack-years,
**Safety during colonisation**	24/7 contact, daily monitoring, emergency antibiotics provided	• 24/7 contact, daily monitoring, emergency antibiotics provided
**Ethical approvals**	Malawi NHSRC, LSTM ethics board	UK National Health Service Research Ethics (12/NW/0873)
**Collaborating laboratories**	LSTM, Public Health England	LSTM, Public Health England

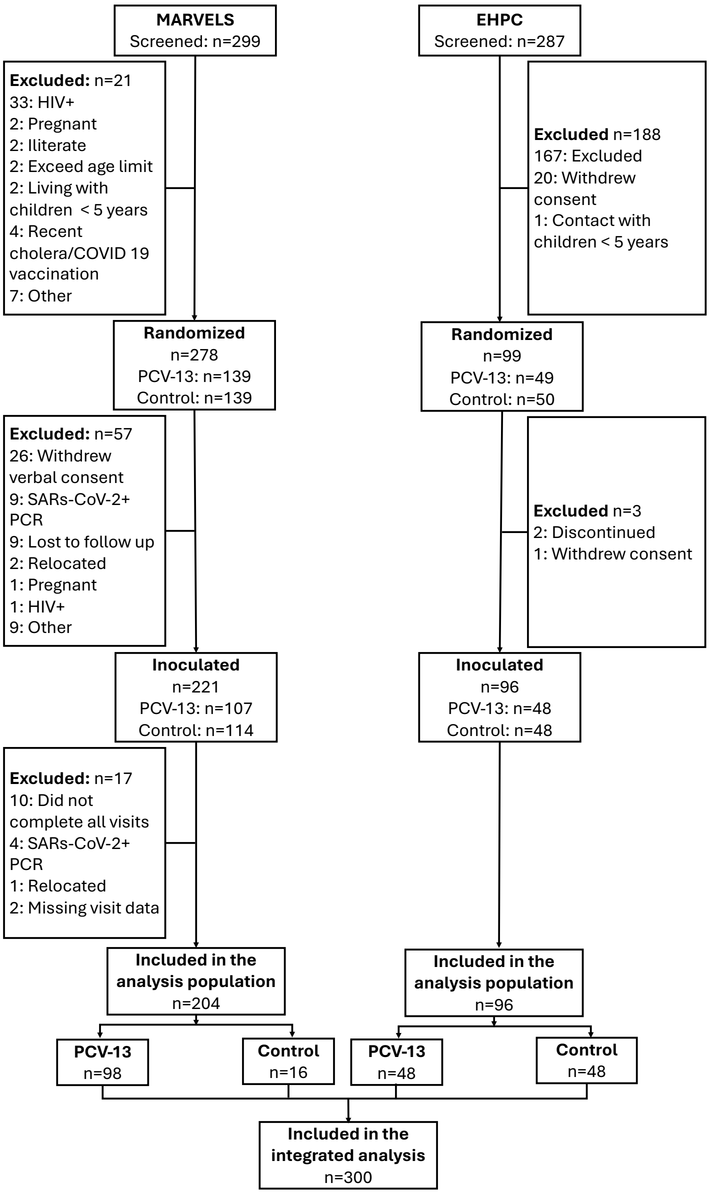

Supplementary Figure 1: CONSORT diagram showing numbers of participants screened, recruited, randomized and analysed from the MARVELS and EHPC trials.

Nasal wash processing and determination of carriage

Nasal wash (NW) sample collection and processing were consistent across both studies, except for the additional (uninformative) day 21 collection in EHPC . Briefly, 5 ml of 0.9% saline was instilled into each naris and held before being allowed to drip into a sterile Galli pot. This process was repeated to obtain a minimum of 10 ml of wash. In the lab, 2 ml of NW was transferred to a 15ml Falcon tube to which 4ml RNAprotect bacterial reagent was added. The mixture was incubated for 5 minutes at room temperature then frozen at -80°C for viral testing later. The remaining NW sample was centrifuged for 10 minutes at 3400Xg. Ten (10) aliquots of the supernatant (1ml each) were stored at -80°C in 10 pre-labelled tubes while the bacterial pellet was resuspended in 100ml of skim milk, tryptone, glucose, and glycerine (STGG). Using the 96-Well, Cell Culture plate, 8 serial dilutions of the pellet were prepared and plated on Columbia sheep blood agar (Oxoid, UK) containing 5μg/ml gentamicin (CBG) to quantify colonisation density. Plates were incubated for 18-24 hours at 37^0C in 5% CO_2, α-haemolytic, draughtsman-like colonies were Gram-stained and sub-cultured for optochin sensitivity and bile solubility. Confirmation of pneumococcal serotypes was performed using latex agglutination [Immulex Pneumotest reagents (Statens Serum Institute, Copenhagen, Denmark)]. Participants in whom experimental pneumococci were detected in NW samples at any visit postinoculation were defined as experimentally colonised. Isolates were frozen at −80°C for storage. All experimentally colonised participants who did not have two consecutive culture-negative NWs received amoxicillin, 500 mg three times per day, for 3 days at the end of the study to ensure 6B colonisation clearance.

Section/topic	No	CONSORT 2025 checklist item description	Reported on page no.
**Title and abstract**			
Title and structured abstract	1a	Identification as a randomised trial	1 and 13
	1b	Structured summary of the trial design, methods, results, and conclusions	1
**Open science**			
Trial registration	2	Name of trial registry, identifying number (with URL) and date of registration	1, 12
Protocol and statistical analysis plan	3	Where the trial protocol and statistical analysis plan can be accessed	13
Data sharing	4	Where and how the individual de-identified participant data (including data dictionary), statistical code and any other materials can be accessed	16
Funding and conflicts of interest	5a	Sources of funding and other support (eg, supply of drugs), and role of funders in the design, conduct, analysis and reporting of the trial	17
	5b	Financial and other conflicts of interest of the manuscript authors	17
**Introduction**			
Background and rationale	6	Scientific background and rationale	1, 2, 3
Objectives	7	Specific objectives related to benefits and harms	1, 3
**Methods**			

Patient and public involvement	8	Details of patient or public involvement in the design, conduct and reporting of the trial	NA
Trial design	9	Description of trial design including type of trial (eg, parallel group, crossover), allocation ratio, and framework (eg, superiority, equivalence, non-inferiority, exploratory)	12, 13
Changes to trial protocol	10	Important changes to the trial after it commenced including any outcomes or analyses that were not prespecified, with reason	12
Trial setting	11	Settings (eg, community, hospital) and locations (eg, countries, sites) where the trial was conducted	12
Eligibility criteria	12a	Eligibility criteria for participants	13
	12b	If applicable, eligibility criteria for sites and for individuals delivering the interventions (eg, surgeons, physiotherapists)	NA
Intervention and comparator	13	Intervention and comparator with sufficient details to allow replication. If relevant, where additional materials describing the intervention and comparator (eg, intervention manual) can be accessed	13
Outcomes	14	Prespecified primary and secondary outcomes, including the specific measurement variable (eg, systolic blood pressure), analysis metric (eg, change from baseline, final value, time to event), method of aggregation (eg, median, proportion), and time point for each outcome	15
Harms	15	How harms were defined and assessed (eg, systematically, non-systematically)	NA
Sample size	16a	How sample size was determined, including all assumptions supporting the sample size calculation	NA
	16b	Explanation of any interim analyses and stopping guidelines	NA
Randomisation:			
Sequence generation	17a	Who generated the random allocation sequence and the method used	13

	17b	Type of randomisation and details of any restriction (eg, stratification, blocking and block size)	13
			**Reported on page no.**
Allocation concealment mechanism	18	Mechanism used to implement the random allocation sequence (eg, central computer/telephone; sequentially numbered, opaque, sealed containers), describing any steps to conceal the sequence until interventions were assigned	NA
Implementation	19	Whether the personnel who enrolled and those who assigned participants to the interventions had access to the random allocation sequence	13
Blinding	20a	Who was blinded after assignment to interventions (eg, participants, care providers, outcome assessors, data analysts)	12-13
	20b	If blinded, how blinding was achieved and description of the similarity of interventions	13
Statistical methods	21a	Statistical methods used to compare groups for primary and secondary outcomes, including harms	15-16
	21b	Definition of who is included in each analysis (eg, all randomised participants), and in which group	15-16
	21c	How missing data were handled in the analysis	14
	21d	Methods for any additional analyses (eg, subgroup and sensitivity analyses), distinguishing prespecified from post hoc	15
**Results**			
Participant flow, including flow diagram	22a	For each group, the numbers of participants who were randomly assigned, received intended intervention, and were analysed for the primary outcome	14-15
	22b	For each group, losses and exclusions after randomisation, together with reasons	NA
Recruitment	23a	Dates defining the periods of recruitment and follow-up for outcomes of benefits and harms	12-14
	23b	If relevant, why the trial ended or was stopped	NA

Intervention and comparator delivery	24a	Intervention and comparator as they were actually administered (eg, where appropriate, who delivered the intervention/comparator, how participants adhered, whether they were delivered as intended (fidelity))	NA
	24b	Concomitant care received during the trial for each group	Supplementary table 3
Baseline data	25	A table showing baseline demographic and clinical characteristics for each group	23
Numbers analysed, outcomes and estimation	26	For each primary and secondary outcome, by group:<br>● the number of participants included in the analysis<br>● the number of participants with available data at the outcome time point<br>● result for each group, and the estimated effect size and its precision (such as 95% confidence interval)<br>● for binary outcomes, presentation of both absolute and relative effect size	4-5, 22-27
Harms	27	All harms or unintended events in each group	NA
Ancillary analyses	28	Any other analyses performed, including subgroup and sensitivity analyses, distinguishing pre-specified from post hoc	16
**Discussion**			
Interpretation	29	Interpretation consistent with results, balancing benefits and harms, and considering other relevant evidence	7-11
Limitations	30	Trial limitations, addressing sources of potential bias, imprecision, generalisability, and, if relevant, multiplicity of analyses	10-11

Citation: Hopewell S, Chan AW, Collins GS, Hróbjartsson A, Moher D, Schulz KF, et al. CONSORT 2025 Statement: updated guideline for reporting randomised trials. BMJ. 2025; 388:e081123. https://dx.doi.org/10.1136/bmj-2024-081123
© 2025 Hopewell et al. This is an Open Access article distributed under the terms of the Creative Commons Attribution License (https://creativecommons.org/licenses/by/4.0/), which permits unrestricted use, distribution, and reproduction in any medium, provided the original work is properly cited.

*We strongly recommend reading this statement in conjunction with the CONSORT 2025 Explanation and Elaboration and/or the CONSORT 2025 Expanded Checklist for important clarifications on all the items. We also recommend reading relevant CONSORT extensions. See www.consort-spirit.org.
